# The Bacterial Translocon SecYEG Opens upon Ribosome Binding*

**DOI:** 10.1074/jbc.M113.477893

**Published:** 2013-05-03

**Authors:** Denis G. Knyazev, Alexander Lents, Eberhard Krause, Nicole Ollinger, Christine Siligan, Daniel Papinski, Lukas Winter, Andreas Horner, Peter Pohl

**Affiliations:** From the ‡ Institute of Biophysics, Johannes Kepler University Linz, A-4020 Linz, Austria and; the § Leibniz Institute for Molecular Pharmacology (FMP), 13125 Berlin, Germany

**Keywords:** Membrane Bilayer, Membrane Biophysics, Membrane Reconstitution, Permeability, Protein Translocation, Channel Gating

## Abstract

In co-translational translocation, the ribosome funnel and the channel of the protein translocation complex SecYEG are aligned. For the nascent chain to enter the channel immediately after synthesis, a yet unidentified signal triggers displacement of the SecYEG sealing plug from the pore. Here, we show that ribosome binding to the resting SecYEG channel triggers this conformational transition. The purified and reconstituted SecYEG channel opens to form a large ion-conducting channel, which has the conductivity of the plug deletion mutant. The number of ion-conducting channels inserted into the planar bilayer per fusion event roughly equals the number of SecYEG channels counted by fluorescence correlation spectroscopy in a single proteoliposome. Thus, the open probability of the channel must be close to unity. To prevent the otherwise lethal proton leak, a closed post-translational conformation of the SecYEG complex bound to a ribosome must exist.

## Teaser

**Background:** How SecYEG opens for co-translational translocation is unknown.

**Results:** Ribosome binding to the SecY complex induces ion channel activity.

**Conclusion:** SecYEG responds to ligand binding by a conformational transition.

**Significance:** Dislocation of the plug prepares entry of the nascent chain.

## Introduction

The heterotrimeric bacterial protein translocation channel SecYEG (translocon or SecY complex) resides in the plasma membrane. It enables many water-soluble proteins to pass into the periplasmic space (1). Its evolutionarily conserved family member, the eukaryotic Sec61 complex, transports proteins from the cytoplasm into the endoplasmic reticulum lumen. Both the SecY and Sec61 complexes also serve to insert hydrophobic proteins through a lateral gate into the surrounding membrane (2). The complexes are understood to be closed between translocation events, thereby preventing proton and calcium leakage. The pore ring, a hydrophobic constriction zone in the middle of the channel, and the plug, a mobile reentrant loop in the periplasmic half of the funnel, seal the resting channel (3).

In post-translational translocation, a gating function is suggested for the nascent chain signal sequence by experiments in which large ion-conducting channels appeared in bacterial membranes upon addition of a synthetic signal peptide (4). Whether the signal peptide of membrane proteins acts similarly is not yet known. Moreover, it is unclear how the hydrophobic part of the signal sequence reaches its binding site between TM2b and TM7. This site is blocked (5) in the resting state of the SecY complex. Intercalation of the signal peptide requires the SecY helices TM2b and TM7 to undergo spontaneous separation (6). Previously, the role of a separation trigger was attributed to the dimerization of the SecY complex in the plane of the membrane because an electron microscopy structure showed the dimeric *Escherichia coli* SecY with a partially open lateral gate (7). However, a recent electron microscopy structure of a reconstituted translocon in a nanodisc (8) and functional single molecule studies of reconstituted translocons in proteoliposomes (9) suggest that dimer formation is not required for protein translocation, *i.e.* even if dimerization would provide access to the signal peptide-binding site, the question of how the monomer opens would still be unsolved.

The most straightforward hypothesis is that the ribosome itself acts as a pore opener upon binding to the translocation channel. However, site-specific labeling with an environment-sensitive fluorophore failed to report plug conformation changes upon binding and insertion of a ribosome-bound nascent membrane protein (10). Moreover, electron microscopy revealed a nearly closed lateral gate of the mammalian translocon in a ribosome-bound conformation (11). Only the most recent structure of the SecY complex (Protein Data Bank code 3J01) pictured the channel with a partially open lateral gate but with the plug still occluding the pore (8). These results do not agree well with electrophysiological experiments performed >20 years ago. Microsomal membranes containing the eukaryotic translocation channel revealed large ion channels that appeared after nascent chain release by puromycin and vanished after translocon-ribosome complex dissociation (12).

The conductance of these channels is roughly similar to the conductance of the plugless SecYEG mutant (3). This observation suggests that a conformation of the ribosome-bound translocon exists in which the channel sealing plug is removed from the pore. Whether the ribosome serves to induce channel opening is unclear. To solve this question, we reconstituted the purified SecY complex into planar lipid bilayers and monitored single channel openings upon ribosome binding.

## MATERIALS AND METHODS

### 

#### 

##### Protein Expression and Purification

The SecY complex was essentially purified as described (13). Mutants were generated by PCR mutagenesis and verified by sequencing. The expression of the SecY complex in C43(DE3) cells was induced with arabinose for 4 h at 37 °C. The membranes were solubilized in 1% dodecyl-β-d-maltopyranoside (Anatrace), and the extract was passed over a Ni^2+^-chelating column. The protein eluted with imidazole was further purified by size-exclusion chromatography (5). Protein concentrations were determined with Bradford reagent (Bio-Rad) or, in case of the labeled mutant, by fluorescence correlation spectroscopy. Purified SecY complexes were stored at −80 °C in 10 mm Tris-Cl (pH 8.0), 150 mm NaCl, 10% glycerol, 10 mm DTT, and 0.03% dodecyl-β-d-maltopyranoside. Bacterial ribosomes were purified from *E. coli* MRE600 as described previously (14, 15) and kindly provided by the Rapoport laboratory.

##### Protein Reconstitution into Lipid Vesicles

The purified SecY complex was reconstituted into proteoliposomes by dialysis. In brief, the reconstitution mixture was prepared at room temperature by sequentially adding 50 mm K-HEPES, 1 mm DTT, 6% (w/v) Deoxy Big CHAP, purified protein (∼100 μg in detergent), and 10 mg of preformed *E. coli* polar phospholipid vesicles (Avanti Polar Lipids, Alabaster, AL). The mixture was placed into Spectra/Por 2.1 dialysis tubing (molecular mass cutoff of 15,000; Spectrum Laboratories, Inc., Laguna Hills, CA) and dialyzed against 100 volumes of assay buffer (50 mm K-HEPES (pH 7.5), 200 mm potassium acetate, 1 mm DTT, 10% glycerol, and protease inhibitor) for 72 h at 4 °C. The proteoliposomes were harvested by ultracentrifugation at 100,000 × *g* for 60 min and resuspended in assay buffer at a concentration of 5–10 mg/ml.

##### Reconstitution of the Closed SecY Complex into Planar Bilayers

In one of the two chambers (called the *cis* chamber) of a Teflon cell, proteoliposomes containing either the wild-type SecY complex at a protein/lipid ratio of ∼1:70 or the mutant SecY complex (F67C/R357E) at a protein/lipid ratio of ∼1:100 were mixed with empty lipid vesicles (*E. coli* polar lipid) to reach a final lipid concentration of 1.3 mg/ml. The second Teflon cell chamber (called the *trans* chamber) hosted empty vesicles at a final concentration of 1.3 mg/ml. The buffer contained 50 mm K-HEPES (pH 7.5), 150 mm KCl, and protease inhibitor mixture.

After lipid monolayers had formed on top of the vesicle suspensions, the level of the buffer solutions was raised above the aperture in the Teflon septum that separated both suspensions. During this procedure, the two monolayers spontaneously combined within the aperture (150–250 μm in diameter) (16, 17). It is important to note that the septum was pretreated with a 1:200 solution of hexadecane in hexane. After a stable membrane had formed, 5 mm MgCl_2_ was added to the *cis* chamber. In the absence of the ligand, control current recordings were undertaken to ensure that lipid channels were not present.

Thereafter, ribosomes (36 μg/μl stock) were added to the *cis* chamber at a final concentration of 0.6–1.2 μg/μl. Some of the experiments were carried out with 0.1–0.2 μg/μl 50 S ribosomal subunits or with 0.1–0.2 μg/μl 30 S subunits.

##### Reconstitution of the Open SecY Complex into Planar Lipid Bilayers by Proteoliposome Fusion

The fusion assay was used for the experiment in Fig. 6 only. The polar lipid extract from *E. coli* was dissolved in hexane. Subsequently, the lipid solution was spread on top of the aqueous phase (50 mm K-HEPES (pH 7.5) and 100 mm KCl) on the *cis* and *trans* sides of the septum to form lipid monolayers (18, 19). After evaporating the solvent, the buffer solution levels in both chambers were raised above the aperture using syringes. The septum was pretreated with a 1:200 solution of hexadecane in hexane. The two monolayers spontaneously combined to a bilayer within the aperture.

After incubation with ribosomes, the proteoliposomes were added to the *cis* chamber, which also contained 5 mm Mg^2+^. The proteoliposomes fused spontaneously (20) with the preformed planar bilayer. After only one or two fusion events, we stopped stirring the bulk solution to inhibit fusion.

##### Single Ion Channel Measurements

Ag/AgCl reference electrodes were immersed in the buffer solutions on both sides of the planar bilayers. The transmembrane current was measured by a patch clamp amplifier (model EPC9, HEKA Electronik) under voltage clamp conditions. The recording filter was a four-pole Bessel with a 3-db corner frequency of 0.1 kHz. The raw data were analyzed using the TAC software package (Bruxton Corp., Seattle, WA). Gaussian filters of 12 Hz were applied to reduce noise.

##### Fluorescence Correlation Spectroscopy

Fluorescence correlation spectroscopy was used to measure channel abundance in the membrane (21). In brief, the average residence time (τ*_D_*) and number of proteoliposomes reconstituted with SecY molecules (labeled with ATTO 488) in the focal volume were derived from the autocorrelation function (*G*(τ)) of the fluorescence temporal signal. The signal was obtained using a commercial laser scanning microscope equipped with avalanche diodes (LSM 510 META/ConfoCor 3, Carl Zeiss, Jena, Germany). We consequently applied the standard model for one-component free three-dimensional diffusion (22). The number of fluorescent particles (*n*) in the detection volume (*V*_eff_) was *n* = *V*_eff_*C*, where *C* is the particle concentration. The diffusion coefficient (*D*) was determined as ω^2^/4τ*_D_*, where ω = 0.16 μm^2^ is the diameter of the confocal volume cross-section. A water drop formed the connection between the 40× water immersion objective and the coverslip, which provided the base of the measurement chamber.

Dissolving the vesicles with Triton X-100 resulted in micelle formation. Due to their smaller size, the τ*_D_* value dropped by ∼10-fold, *i.e.* from ∼2 ms to 200 μs. The number of micelles per confocal volume divided by the number of proteoliposomes, *i.e.* the particle number before micellation, was taken as the number of SecY complexes per vesicle.

##### Labeling of the SecY Complex

The SecY(A204C)-containing extract was passed over a Ni^2+^-chelating column, concentrated, and incubated with tris(2-carboxyethyl)phosphine (Fluka) for 5 min at 4 °C. ATTO 488-maleimide (100 μm) was added and stored overnight under steady mixing at 4 °C. The sample was diluted with solubilization buffer (300 mm NaCl, 0.6 mm dodecyl-β-d-maltopyranoside, 10% glycerol, and 20 mm Tris (pH 7.5)) to reduce the imidazole concentration to <10 mm and was passed over a Ni2+-chelating column. The protein eluted with imidazole was further purified by size-exclusion chromatography and reconstituted as described above.

##### Mass Spectrometry

LC-MS measurements were performed as described (23). In brief, proteins were digested with trypsin (sequencing grade; Promega) or Asp-N (sequencing grade; Roche Diagnostics) according to the standard protocol. Peptides were analyzed using a reversed-phase capillary liquid chromatography system (Eksigent NanoLC-Ultra, Axel Semrau GmbH & Co. KG) connected to an LTQ Orbitrap XL mass spectrometer (Thermo Scientific). LC separations were performed on a PepMap100 C18 capillary column (3 μm, 100 Å, 250 mm × 75 μm (inner diameter); Dionex) at an eluent flow rate of 300 nl/min using a linear gradient of 4–60% mobile phase B (0.1% formic acid in acetonitrile) for 70 min. Mobile phase A contained 0.1% formic acid in water. Mass spectra were acquired in a data-dependent mode with one MS survey scan (with a resolution of 60,000) in the Orbitrap spectrometer and with MS/MS scans of the five most intense precursor ions in the linear trap quadrupole. The processed MS/MS data and MASCOT server (version 2.0, Matrix Science Ltd., London, United Kingdom) were used to search in-house against the Swiss-Prot database (version 2010_10, Taxonomy, *E. coli* 22708 sequences). The mass tolerance of precursor and sequence ions was set to 10 ppm and 0.35 Da, respectively. Methionine oxidation and the acrylamide modification of cysteine were used as variable modifications.

## RESULTS

**FIGURE 1.**
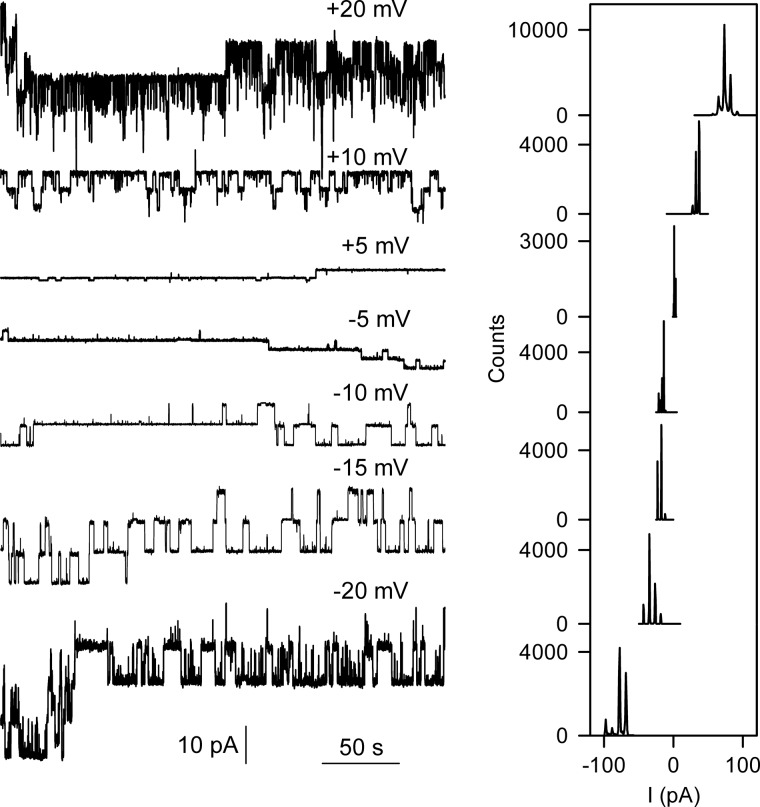
**Single channel activity of the wild-type SecY channel induced by ribosome binding.** *Left panel*, single channel traces at different voltages. *Right panel*, the corresponding amplitude histograms.

We purified the protein-conducting SecY complex from *E. coli* and reconstituted it into liposomes made from *E. coli* polar phospholipids. The presence of the wild-type SecY complex in the bilayer did not increase the membrane conductivity compared with empty bilayers. It was only after the addition of purified *E. coli* ribosomes that long-lasting channel openings were recorded (Fig. 1). Histogram analysis revealed a voltage-independent *g* value of 530 picosiemens in 150 mm KCl. This is somewhat smaller than expected from mutants with full plug deletion (Δ60–74) but larger than measured for mutants with partial plug deletion (Δ65–70) (3).

**FIGURE 2.**
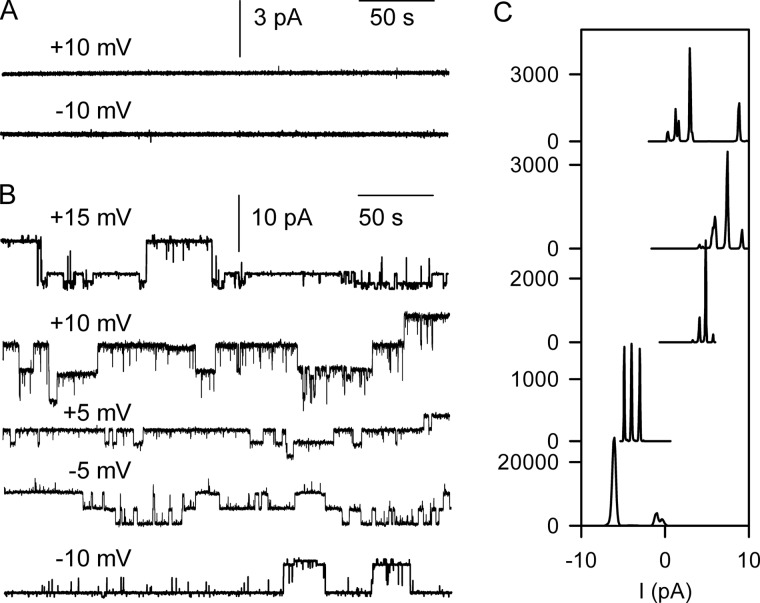
**Channel activation by isolated ribosomal subunits.** *A*, the isolated 30 S subunit was unable to activate wild-type SecY. *B*, channel activity was observed only upon subsequent or sole addition of isolated 50 S subunits to the same membrane. Representative single channel traces at different voltages are shown. *C*, amplitude histograms corresponding to the recordings in *B*.

The SecY channel is known to interact with the 50 S ribosomal subunit (15, 24). We performed experiments with the isolated 30 S subunit to confirm specific binding. We did not observe gating in its presence (Fig. 2*A*). Channel opening was observed exclusively when 30 S subunit addition was followed by 50 S subunit addition. It was also detected when just 50 S subunits were added (Fig. 2*B*). Histogram analysis revealed that the amplitudes of channels induced by whole ribosomes or 50 S subunits were identical (Figs. 1 and 2*C*). The addition of ribosomes or the 50 S subunit in the absence of the reconstituted SecY complex did not result in channel activity.

**FIGURE 3.**
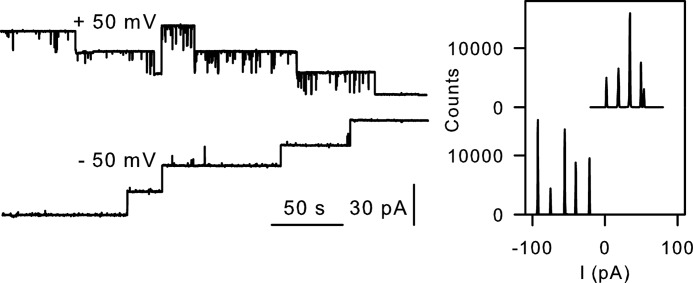
**Point mutation R357E does not inhibit ribosome binding.** *Left panel*, ribosome-induced single channel activity observed with the double mutant SecY complex (F67C/R357E). *Right panel*, the corresponding amplitude histograms.

Biochemical evidence suggested that ribosome binding to the SecY complex is impaired by the SecY point mutation R357E (25). To test this hypothesis, we introduced a second mutation (F67C). Flickering of the reconstituted channel upon tetrathionate binding to that cysteine (3) allows verification of channel reconstitution when the ribosome fails to activate the channel. However, we observed gating of the double mutant after ribosome addition (Fig. 3). Channel openings lasted from several seconds up to minutes. The only difference from the wild-type channel was the smaller *g* value of the double mutant. It was most probably due to the presence of the additional cysteine at position 67. We cannot exclude the possibility that a partial reduction in binding probability may have escaped our attention because instead of analyzing the whole ensemble of events, we performed only single channel analysis.

**FIGURE 4.**
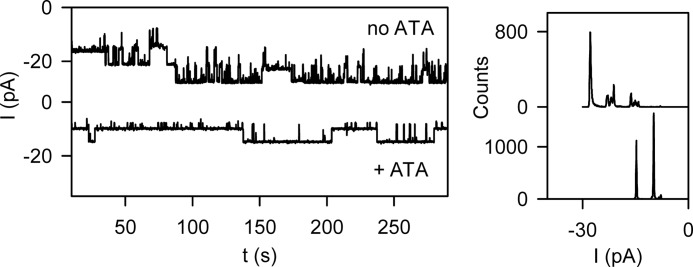
**Partial inhibition of ribosome binding by ATA.** *Left panel*, the *upper trace* shows the simultaneous openings of five wild-type translocation channels. The addition of ATA reduced the number of open channels to two (*lower trace*), indicating that ATA partially inhibits ribosome binding. *Right panel*, the corresponding amplitude histograms.

Ribosome dissociation from the SecY complex is known to be accelerated by aurintricarboxylic acid (ATA).^4^ ATA leaves ribosomes intact and is expected to prevent the reformation of SecYEG-ribosome bonds (26, 27). The recording shown in Fig. 4 is representative of a dozen independent experiments that revealed that ATA reduced the number of open channels: in this case, from five open channels (*upper trace*) to two open channels (*lower trace*). An inhibition efficiency of 60% agrees well with the results obtained from biochemical assays (28).

**FIGURE 5.**
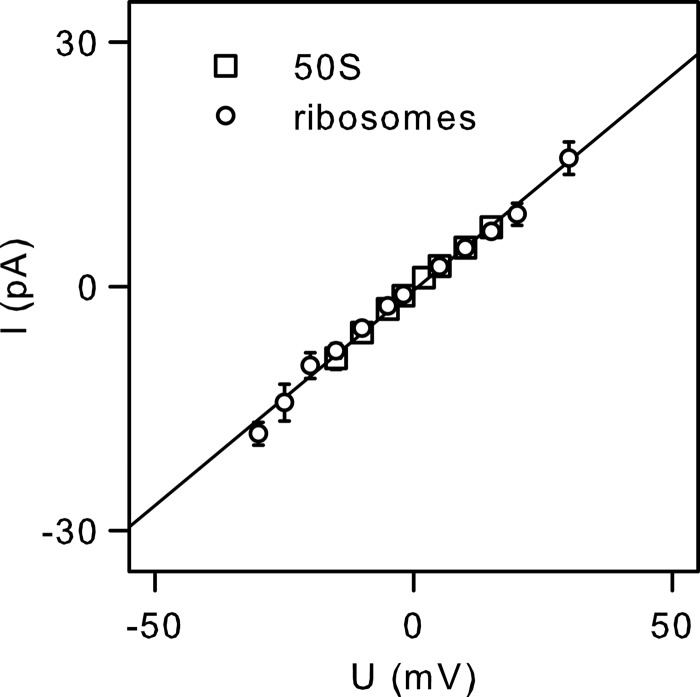
**Current-voltage characteristics of single SecY complexes activated by whole ribosomes and the 50 S ribosomal subunit.** The channel amplitude is highly reproducible. *Error bars* indicate S.D. and are not shown if they were smaller than the symbol. The slope of the linear regression corresponds to a single channel conductance (*g*) of ∼532 picosiemens.

The conformational transitions induced by ribosomes or by 50 S subunits are highly reproducible. We observed single channel current linear dependence on transmembrane voltage (Fig. 5). If our ribosome preparation had contained a small amount of proteins with uncleaved signal peptides and if these signal peptides had been responsible for the opening of the SecY complex, we would have expected variable channel amplitudes. This is what was observed upon binding of the OmpA (outer membrane protein A) precursor protein signal peptide to the reconstituted SecY complex.^5^ The possibility that we mixed actual ribosome-induced channel openings with signal peptide-induced openings is thus rather unlikely.

To exclude the small remaining probability, we analyzed our ribosome preparation by MS. To identify proteins with a signal sequence at their N terminus, which may have primed them for the SecYEG pathway, ribosome samples were digested with either trypsin or Asp-N, and resulting peptides were subsequently subjected to LC-MS/MS. According to Tjalsma *et al.* (30), these proteins should have had a positively charged N terminus, followed by a hydrophobic span and a signal peptidase cleavage site (A*X*A … A). Using fragment ion spectra (MS/MS) of identified peptides resulting from both trypsin cleavage (Lys- and Arg-specific) and Asn-specific cleavage by an endoproteinase, we performed an unbiased search against the Swiss-Prot protein database for the presence of proteins with signal peptide sequences (data not shown). Because we were unable to detect any signal peptide sequences by MS, we concluded that the observed openings of the SecY complex must have been induced by ribosomes.

**FIGURE 6.**
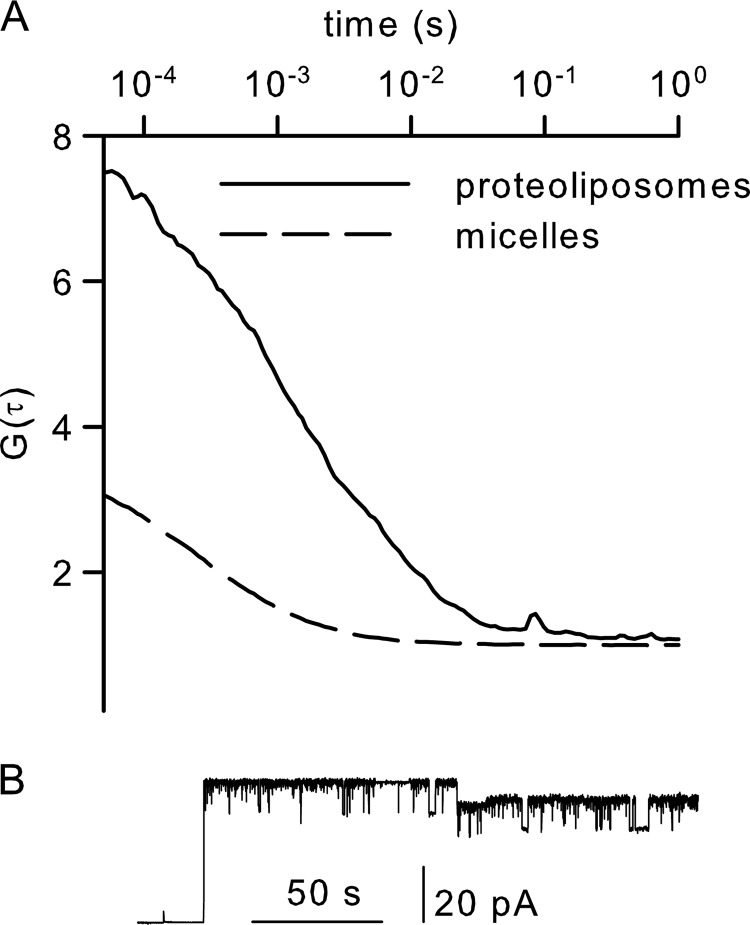
*A*, probability of SecYEG to open upon ribosome binding. A suspension of proteoliposomes containing the ATTO 488-labeled SecY(A204C) complex was measured by fluorescence correlation spectroscopy. The autocorrelation function (*G*(τ)) indicated that, on average, 0.15 vesicles were in the confocal volume. Dissolving the vesicles by detergent increased the particle number to 0.5. The 0.15/0.5 ratio indicated that every vesicle contained approximately three copies of the SecY complex. *B*, in the presence of ribosomes, fusion of these vesicles with preformed planar bilayers resulted in channel activity. The initial current jump indicates the fusion of a single vesicle, which contained four copies of the SecY complex, as derived from the ratio of the initial current jump to the single channel amplitude.

Channel opening by ribosomes is at odds with *in vivo* experiments in which the translocon was found to be impermeable to ions (31). We figured that a low open probability of <0.001 could explain this discrepancy because a single *E. coli* bacterium usually possesses about ∼500 copies of the translocon (32). To test this hypothesis, we changed the reconstitution procedure. Instead of folding bilayers from monolayers, we fused proteoliposomes with preformed bilayers. After each fusion event, a stepwise increase in the number of open channels in the planar bilayer was observed (Fig. 6).

To determine whether the increment in channel number matched the number of individual translocons in the proteoliposome, we subjected a small number of vesicles to fluorescence correlation spectroscopy. To this end, we introduced a cysteine at position 204 of SecY and labeled it by conjugation to the fluorescent dye ATTO 488. We established the number of channels per proteoliposome in a two-step procedure. We first measured the vesicle number in the proteoliposome suspension. We then dissolved the vesicles by detergent addition and determined the number of labeled particles again. Assuming that every micelle contained exactly one channel, the ratio of fluorescent micelles to vesicles indicated the number of SecY complexes per vesicle (Fig. 6*A*). This number was in reasonable agreement with the number of channels introduced into the planar bilayer per fusion event. This observation suggested an open probability of close to 1 (Fig. 6*B*).

## DISCUSSION

We have shown that ribosome binding is sufficient to open the SecY complex. The experimentally established similarity in the *g* values of the open channel (Fig. 5) and the plug deletion mutants (3) indicates that this ligand is able to fully open the channel. Furthermore, the *g* value agreed reasonably well with that previously ascribed to the translocation complex subsequent to release of the nascent chain (12). This observation is particularly interesting because similar *g* values for the prokaryotic and eukaryotic translocons (4, 33) are in line with the conservation of the translocon amino acid sequence and its function. Apparently, the recently observed movement of the lateral gate *in silico* appeared to be very slight only because of the short simulation time of 1–2 μs (34).

The new role of the ribosome as a channel activator reverses its previously assumed role as a gate keeper that tightly shielded the nascent chain from the cytoplasm (35). In the eukaryotic system, the ribosome was reported to seal the translocon from the cytoplasm so efficiently that it prevented a fluorescent dye on the nascent chain to be quenched by small molecules (35). At present, we cannot entirely rule out that the eukaryotic translocon is fundamentally different from the prokaryotic one. Thus, caution is required when extrapolating our conductivity data from the SecY-ribosome complex to the Sec61-ribosome complex. Indeed, a series of electrophysiological experiments suggests that the Sec61 complex is an intrinsically open channel (36, 37), much in contrast to the intrinsically closed SecY complex (3). However, conserved structural features, such as the hydrophobic pore ring, the plug, and the lateral gate (8, 11, 38), argue against a fundamentally different translocation mechanism.

In the resting state, the plug not only seals the channel, its interaction with neighboring amino acids also acts to stabilize the pore ring. Accordingly, the removal of the plug not only enables ion conductance, it also allows the pore ring to widen and the lateral gate to open. This would explain how the signal peptide reaches its binding site during co-translational translocation, which is occluded in the crystal structure (5). After translocation has been completed, the translocon may revert to its closed state while the ribosome is still bound. Conceivably, this is the state that was captured by cryo-electron microscopy (11). A closed ribosome-bound conformation would also explain the viability of plug deletion mutants (29).

We conclude that ribosome binding is sufficient to open the translocon. The opening may prepare the channel for signal peptide binding. The transition of SecY to a closed ribosome-bound conformation remains to be shown, as well as the lack of proton permeability with the nascent chain inserted into the channel.

## Footnotes

5 D. G. Knyazev, L. Winter, N. Ollinger, C. Siligan, and P. Pohl, submitted for publication.

## Footnotes

4 The abbreviation used is:

ATA: aurintricarboxylic acid.

## References

[1] Driessen A. J. Nouwen N. (2008) Protein translocation across the bacterial cytoplasmic membrane. Annu. Rev. Biochem. 77, 643–667 18078384 10.1146/annurev.biochem.77.061606.160747

[2] Rapoport T. A. (2007) Protein translocation across the eukaryotic endoplasmic reticulum and bacterial plasma membranes. Nature 450, 663–669 18046402 10.1038/nature06384

[3] Saparov S. M. Erlandson K. Cannon K. Schaletzky J. Schulman S. Rapoport T. A. Pohl P. (2007) Determining the conductance of the SecY protein translocation channel for small molecules. Mol. Cell 26, 501–509 17531809 10.1016/j.molcel.2007.03.022

[4] Simon S. M. Blobel G. (1992) Signal peptides open protein-conducting channels in *E. coli*. Cell 69, 677–684 1375130 10.1016/0092-8674(92)90231-z

[5] van den Berg B. Clemons W. M. Jr. Collinson I. Modis Y. Hartmann E. Harrison S. C. Rapoport T. A. (2004) X-ray structure of a protein-conducting channel. Nature 427, 36–44 14661030 10.1038/nature02218

[6] Li W. Schulman S. Boyd D. Erlandson K. Beckwith J. Rapoport T. A. (2007) The plug domain of the SecY protein stabilizes the closed state of the translocation channel and maintains a membrane seal. Mol. Cell 26, 511–521 17531810 10.1016/j.molcel.2007.05.002

[7] Bostina M. Mohsin B. Kühlbrandt W. Collinson I. (2005) Atomic model of the *E. coli* membrane-bound protein translocation complex SecYEG. J. Mol. Biol. 352, 1035–1043 16154141 10.1016/j.jmb.2005.08.005

[8] Frauenfeld J. Gumbart J. van der Sluis E. O. Funes S. Gartmann M. Beatrix B. Mielke T. Berninghausen O. Becker T. Schulten K. Beckmann R. (2011) Cryo-EM structure of the ribosome-SecYE complex in the membrane environment. Nat. Struct. Mol. Biol. 18, 614–621 21499241 10.1038/nsmb.2026 PMC3412285

[9] Kusters I. van den Bogaart G. Kedrov A. Krasnikov V. Fulyani F. Poolman B. Driessen A. J. M. (2011) Quaternary structure of SecA in solution and bound to SecYEG probed at the single molecule level. Structure 19, 430–439 21397193 10.1016/j.str.2010.12.016

[10] Lycklama a Nijeholt J. A. Wu Z. C. Driessen A. J. M. (2011) Conformational dynamics of the plug domain of the SecYEG protein-conducting channel. J. Biol. Chem. 286, 43881–43890 22033919 10.1074/jbc.M111.297507 PMC3243504

[11] Becker T. Bhushan S. Jarasch A. Armache J. P. Funes S. Jossinet F. Gumbart J. Mielke T. Berninghausen O. Schulten K. Westhof E. Gilmore R. Mandon E. C. Beckmann R. (2009) Structure of monomeric yeast and mammalian Sec61 complexes interacting with the translating ribosome. Science 326, 1369–1373 19933108 10.1126/science.1178535 PMC2920595

[12] Simon S. M. Blobel G. (1991) A protein-conducting channel in the endoplasmic reticulum. Cell 65, 371–380 1902142 10.1016/0092-8674(91)90455-8

[13] Cannon K. S. Or E. Clemons W. M. Jr. Shibata Y. Rapoport T. A. (2005) Disulfide bridge formation between SecY and a translocating polypeptide localizes the translocation pore to the center of SecY. J. Cell Biol. 169, 219–225 15851514 10.1083/jcb.200412019 PMC2171872

[14] Clemons W. M. Jr. Brodersen D. E. McCutcheon J. P. May J. L. Carter A. P. Morgan-Warren R. J. Wimberly B. T. Ramakrishnan V. (2001) Crystal structure of the 30 S ribosomal subunit from *Thermus thermophilus*: purification, crystallization and structure determination. J. Mol. Biol. 310, 827–843 11453691 10.1006/jmbi.2001.4778

[15] Ménétret J. F. Schaletzky J. Clemons W. M. Jr. Osborne A. R. Skånland S. S. Denison C. Gygi S. P. Kirkpatrick D. S. Park E. Ludtke S. J. Rapoport T. A. Akey C. W. (2007) Ribosome binding of a single copy of the SecY complex: implications for protein translocation. Mol. Cell 28, 1083–1092 18158904 10.1016/j.molcel.2007.10.034

[16] Pohl P. Saparov S. M. Borgnia M. J. Agre P. (2001) Highly selective water channel activity measured by voltage clamp: analysis of planar lipid bilayers reconstituted with purified AqpZ. Proc. Natl. Acad. Sci. U.S.A. 98, 9624–9629 11493683 10.1073/pnas.161299398 PMC55502

[17] Saparov S. M. Kozono D. Rothe U. Agre P. Pohl P. (2001) Water and ion permeation of aquaporin-1 in planar lipid bilayers. Major differences in structural determinants and stoichiometry. J. Biol. Chem. 276, 31515–31520 11410596 10.1074/jbc.M104267200

[18] Montal M. Mueller P. (1972) Formation of bimolecular membranes from lipid monolayers and a study of their electrical properties. Proc. Natl. Acad. Sci. U.S.A. 69, 3561–3566 4509315 10.1073/pnas.69.12.3561 PMC389821

[19] Krylov A. V. Pohl P. Zeidel M. L. Hill W. G. (2001) Water permeability of asymmetric planar lipid bilayers: leaflets of different composition offer independent and additive resistances to permeation. J. Gen. Physiol. 118, 333–340 11585847 10.1085/jgp.118.4.333 PMC2233699

[20] Miller C. (1982) Coupling of water and ion fluxes in a K^+^-selective channel of sarcoplasmic reticulum. Biophys. J. 38, 227–230 6285998 10.1016/S0006-3495(82)84552-9 PMC1328862

[21] Erokhova L. Horner A. Kügler P. Pohl P. (2011) Monitoring single-channel water permeability in polarized cells. J. Biol. Chem. 286, 39926–39932 21940624 10.1074/jbc.M111.291864 PMC3220579

[22] Magde D. Elson E. L. Webb W. W. (1974) Fluorescence correlation spectroscopy. II. An experimental realization. Biopolymers 13, 29–61 4818131 10.1002/bip.1974.360130103

[23] Lange S. Sylvester M. Schümann M. Freund C. Krause E. (2010) Identification of phosphorylation-dependent interaction partners of the adapter protein ADAP using quantitative mass spectrometry: SILAC *vs* ^18^O-labeling. J. Proteome Res. 9, 4113–4122 20568816 10.1021/pr1003054

[24] Gumbart J. Trabuco L. G. Schreiner E. Villa E. Schulten K. (2009) Regulation of the protein-conducting channel by a bound ribosome. Structure 17, 1453–1464 19913480 10.1016/j.str.2009.09.010 PMC2778611

[25] Mori H. Ito K. (2001) An essential amino acid residue in the protein translocation channel revealed by targeted random mutagenesis of SecY. Proc. Natl. Acad. Sci. U.S.A. 98, 5128–5133 11309495 10.1073/pnas.081617398 PMC33175

[26] Borgese N. Mok W. Kreibich G. Sabatini D. D. (1974) Ribosomal-membrane interaction: *in vitro* binding of ribosomes to microsomal membranes. J. Mol. Biol. 88, 559–580 4449120 10.1016/0022-2836(74)90408-2

[27] Fresno M. Carrasco L. Vazquez D. (1976) Initiation of the polypeptide chain by reticulocyte cell-free systems. Survey of different inhibitors of translation. Eur. J. Biochem. 68, 355–364 976261 10.1111/j.1432-1033.1976.tb10822.x

[28] Schaletzky J. Rapoport T. A. (2006) Ribosome binding to and dissociation from translocation sites of the endoplasmic reticulum membrane. Mol. Biol. Cell 17, 3860–3869 16822833 10.1091/mbc.E06-05-0439 PMC1593163

[29] Junne T. Schwede T. Goder V. Spiess M. (2006) The plug domain of yeast Sec61p is important for efficient protein translocation, but is not essential for cell viability. Mol. Biol. Cell 17, 4063–4068 16822836 10.1091/mbc.E06-03-0200 PMC1556385

[30] Tjalsma H. Bolhuis A. Jongbloed J. D. Bron S. van Dijl J. M. (2000) Signal peptide-dependent protein transport in *Bacillus subtilis*: a genome-based survey of the secretome. Microbiol. Mol. Biol. Rev. 64, 515–547 10974125 10.1128/mmbr.64.3.515-547.2000 PMC99003

[31] Park E. Rapoport T. A. (2011) Preserving the membrane barrier for small molecules during bacterial protein translocation. Nature 473, 239–242 21562565 10.1038/nature10014 PMC3093665

[32] Matsuyama S. Fujita Y. Sagara K. Mizushima S. (1992) Overproduction, purification and characterization of SecD and SecF, integral membrane components of the protein translocation machinery of *Escherichia coli*. Biochim. Biophys Acta 1122, 77–84 1633199 10.1016/0167-4838(92)90130-6

[33] Simon S. M. Blobel G. Zimmerberg J. (1989) Large aqueous channels in membrane vesicles derived from the rough endoplasmic reticulum of canine pancreas or the plasma membrane of *Escherichia coli*. Proc. Natl. Acad. Sci. U.S.A. 86, 6176–6180 2474828 10.1073/pnas.86.16.6176 PMC297800

[34] Gumbart J. C. Teo I. Roux B. Schulten K. (2013) Reconciling the roles of kinetic and thermodynamic factors in membrane-protein insertion. J. Am. Chem. Soc. 135, 2291–2297 23298280 10.1021/ja310777k PMC3573731

[35] Crowley K. S. Liao S. Worrell V. E. Reinhart G. D. Johnson A. E. (1994) Secretory proteins move through the endoplasmic reticulum membrane via an aqueous, gated pore. Cell 78, 461–471 8062388 10.1016/0092-8674(94)90424-3

[36] Wonderlin W. F. (2009) Constitutive, translation-independent opening of the protein-conducting channel in the endoplasmic reticulum. Pflugers Arch. 457, 917–930 18604553 10.1007/s00424-008-0545-y

[37] Erdmann F. Jung M. Maurer P. Harsman A. Zimmermann R. Wagner R. (2010) The mammalian and yeast translocon complexes comprise a characteristic Sec61 channel. Biochem. Biophys. Res. Commun. 396, 714–720 20450886 10.1016/j.bbrc.2010.04.168

[38] Seidelt B. Innis C. A. Wilson D. N. Gartmann M. Armache J. P. Villa E. Trabuco L. G. Becker T. Mielke T. Schulten K. Steitz T. A. Beckmann R. (2009) Structural insight into nascent polypeptide chain-mediated translational stalling. Science 326, 1412–1415 19933110 10.1126/science.1177662 PMC2920484

